# Management of atrial fibrillation for older people with frailty: a systematic review and meta-analysis

**DOI:** 10.1093/ageing/afy180

**Published:** 2018-11-15

**Authors:** Chris Wilkinson, Oliver Todd, Andrew Clegg, Chris P Gale, Marlous Hall

**Affiliations:** 1 Department of Clinical and Population Sciences, Leeds Institute of Cardiovascular and Metabolic Medicine, University of Leeds, Worsley Building, Level 11, Clarendon Way, Leeds, UK; 2 York Teaching Hospital NHS Foundation Trust, Wiggington Road, York, UK; 3 Academic Unit of Eldery Care and Rehabilitation, Bradford Institute for Health Research, University of Leeds, Bradford Royal Infirmary, Duckworth Lane, Bradford, UK

**Keywords:** atrial fibrillation, frailty, anticoagulation, systematic review, older people

## Abstract

**Background:**

despite a large and growing population of older people with frailty and atrial fibrillation (AF), there is a lack of guidance on optimal AF management in this high-risk group.

**Objective:**

to synthesise the existing evidence base on the association between frailty, AF and clinical outcomes.

**Methods:**

a systematic review of studies examining the association between validated measures of frailty, AF and clinical outcomes, and meta-analysis of the association between frailty and oral anticoagulation (OAC) prescription.

**Results:**

twenty studies (30,883 patients) were included, all observational. Fifteen were in hospital, four in the community, one in nursing care. Risk of bias was low-to-moderate. AF prevalence was 3%–38%. In people with AF, frailty was associated with increased stroke incidence, all-cause mortality, symptom severity and length of hospital stay.

Meta-analysis of six studies showed frailty was associated with decreased OAC prescription at hospital admission (pooled adjusted OR 0.45 [95%CI 0.22–0.93], three studies), but not at discharge (pooled adjusted OR 0.40 [95%CI 0.13–1.23], three studies). A community-based study showed increased OAC prescription associated with frailty (OR 2.33 [95%CI 1.03–5.23]).

**Conclusion:**

frailty is common, and associated with adverse clinical outcomes in patients with AF. There is evidence of an association between frailty status and OAC prescription, with different direction of effect in community compared with hospital cohorts. Despite the majority of care for older people being provided in the community, there is a lack of evidence on the association between frailty, AF, anticoagulation and clinical outcomes to guide optimal care in this setting.

## Key points

- Older people with frailty and AF are at risk of worse clinical outcomes.
- Anticoagulation of older people with frailty and AF is an under-researched area.
- Frailty is associated with lower rates of anticoagulation in patients with AF who are admitted to hospital.

## Introduction

The prevalence of atrial fibrillation (AF) increases with age, affecting up to 4.2% of those aged 60–70 years and 17% of those aged 80 years or older [**1**]. Around one in four hospitalised older people have AF [2], so management of AF in older people is a commonly encountered clinical challenge. Old age is a risk factor for thromboembolic outcomes of AF, but there is evidence for a risk-treatment paradox whereby older patients who are at highest risk of complications of AF, including stroke, are not more likely to be prescribed oral anticoagulation (OAC) [3–**6**]. This appears to be related to fear of iatrogenic harm and a lack of confidence in the evidence of benefit in an older population [**7, 8**].

It is increasingly recognised that frailty is a more useful approach to guide care in older people than chronological age. It is a condition characterised by loss of biological reserves, failure of homeostatic mechanisms and vulnerability to a range of adverse outcomes [9], and can help guide more individualised treatments with advancing multi-morbidity and polypharmacy [10]. The prevalence of patients with frailty and AF is growing [**11**], making optimal management an important goal for older people, clinicians, health services and social care [12–14].

National Institute for Health and Care Excellence (NICE) guidance recommends using the CHA_2_DS_2_-VASc score to identify individuals with a high ischaemic stroke risk, and offering OAC to men with a score of 1, and to men or women with a score of 2 or above [**15**]. However, the studies on which the guidance was based did not explicitly assess frailty. Assessment and modification of bleeding risk factors using the HAS-BLED score is recommended, but there may be additional considerations in a population with frailty such as a higher risk of bleeding and falls [**16**]. The optimal treatment strategy for people with AF and frailty is therefore unclear, as there is evidence of increased risk of stroke and of treatment related harms. Whilst direct oral anticoagulants (DOAC) now provide further therapeutic options, generalisability of trial evidence across the spectrum of older people may be limited as they excluded people anticipated to be in the last one to two years of life and those with several co-morbidities [**17–20**].

The objective of this review is to synthesise the existing evidence base on the association between frailty, AF and clinical outcomes, with a particular focus on OAC.

## Methods

The review was conducted according to meta-analysis of observational studies in epidemiology (MOOSE) guidelines, and reported using Preferred Reporting Items for Systematic Reviews and Meta-Analyses (PRISMA) recommendations [**21, 22**].

### Protocol and registration

The review protocol is registered with PROSPERO (CRD42018092951) [**23**].

### Eligibility criteria

Studies that used a validated measure to identify frailty in populations with AF (permanent, paroxysmal or persistent) or atrial flutter were considered eligible. Reviews, case reports, case series and conference proceedings were excluded. Studies were limited to those in the English language.

### Information sources

We searched CINAHL, Cochrane, Embase, Medline and Web of Science from inception of each until October 2017. The search strategy was developed with a research librarian (Supplementary data, available in *Age and Ageing* online).

### Study selection

Two independent reviewers (C.W. and O.T.) screened titles and abstracts for potentially eligible studies, and assessed full-text articles against the eligibility criteria. All disagreements were resolved through consensus. Reasons for exclusion of articles at the full-text review stage were collated using Covidence [**24**].

### Data extraction

Data from the included studies was extracted using a pro forma including author, year of publication, study period, study design, country, setting, patient characteristics (age, sex, prevalence of co-morbidities, ethnicity), frailty measure, AF prevalence and outcomes assessed. Where frailty status was dichotomised, the threshold used by the study author was used. Data for meta-analysis were extracted by two independent reviewers (C.W. and O.T.).

### Outcomes

The primary outcome was OAC prescription by frailty status. Secondary outcomes included: ischaemic and haemorrhagic stroke; all-cause mortality; disability; care home admission; hospitalisation and haemorrhagic events.

### Risk of bias in individual studies

The Newcastle–Ottawa checklist was used by two authors (C.W. and O.T.) to independently assess risk of bias [**25, 26**], with an adapted scale for cross-sectional studies [**27**]. Studies were assessed on the domains of selection, comparability, exposure and outcome. Studies rated as moderate or good were considered as having low risk of bias.

### Synthesis of results

Two authors (C.W. and O.T.) extracted adjusted odds ratios (ORs) with 95% CIs for dichotomous data. OR for frail versus non-frail were used; when the reverse was reported by the authors then an inverse OR was calculated. We synthesised data for meta-analysis by generic inverse variance random-effects modelling summarised as an OR using RevMan 5.3 software [**28**]. Random-effects modelling was selected because we anticipated that the classification of frailty status may be based on different instruments, and to allow for clinical heterogeneity. Adjusted data were prioritised because they account for confounding variables and are considered more reliable. Unadjusted ORs were not included in the meta-analysis.

## Results

### Study selection

**Figure 1.**
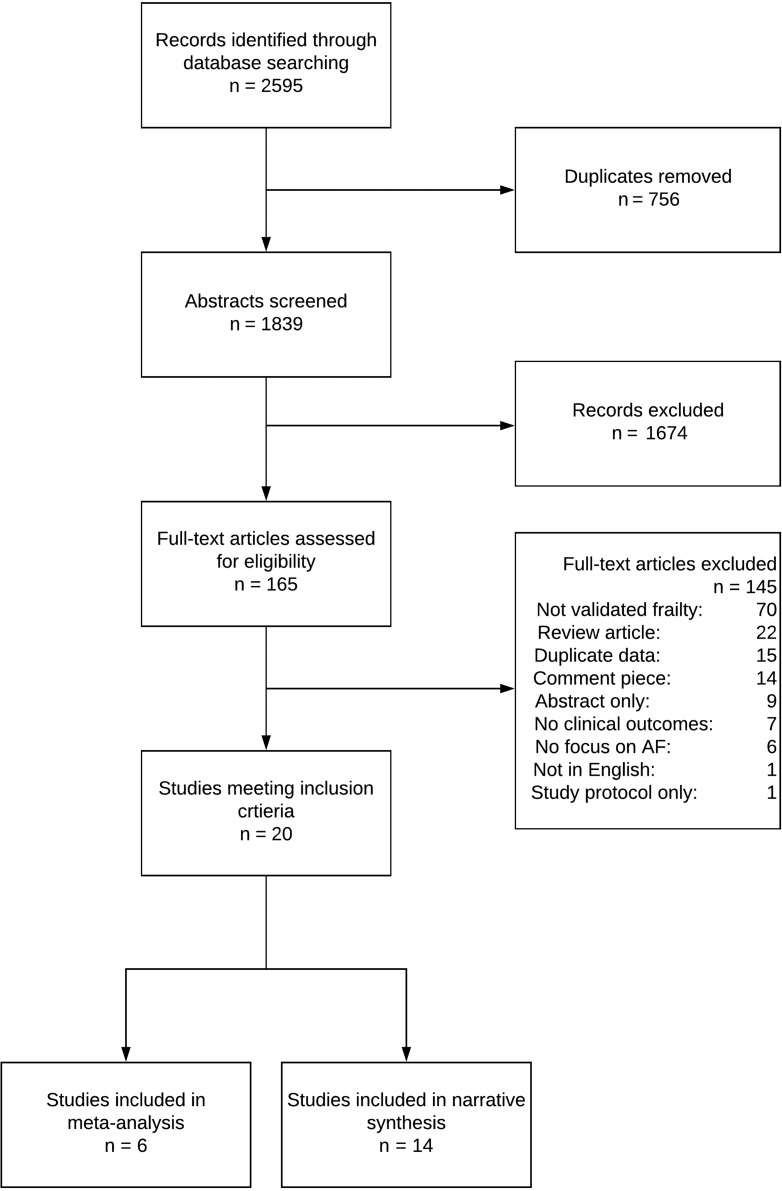
PRISMA diagram of included studies

The review is summarised in Figure 1. The search identified 1,839 studies, of which 165 were retrieved for full-text review. Of these, 20 met the eligibility criteria and are included in this review; 6 within a meta-analysis [3, 29–33] and 14 in a narrative synthesis [2, 34–46]. All were observational studies.

### Study characteristics

Twelve cross-sectional [2, 29–31, 33–40] and eight cohort studies were included [3, 32, 41–46], with a total of 30,883 participants (Table 1). Fifteen studies were based in hospital [2, 3, 29–32, 34, 36, 37, 39, 41–43, 45, 46], and five were community-based [33, 35, 38, 40, 44], one of which involved nursing home residents [38]. Thirteen studies were conducted in Europe [2, 30, 31, 33–35, 37–39, 41–43, 46], three in Australia [32, 38, 45], three in North America [29, 40, 44] and one in Taiwan [36].

**Table 1.** Summary of included studies

Study	Setting	Age criteria	Mean [median] age	Country	Measure of frailty	*n*	Overall risk of bias
Prospective cross-sectional studies							
Bo (2015) [31]	Hospital	≥65	81.7	Italy	GFI	513	Low
Denoël (2014) [34]	Hospital	≥75	NR	Belgium	ISAR	995	Low
Donoghue (2014) [35]	Community	≥50	70.7	Republic of Ireland	GU&G, Gait speed	4,525	Low
Frewen (2013) [47]	Community	≥50	63.8	Republic of Ireland	Fried criteria	4,890	Low
Hess (2013) [40]	Outpatients	≥18	[**75**]	The USA	Fried criteria	10,096	Low
Hung (2013) [36]	Hospital	≥75	[**75**]	Taiwan	GU&G	401	Low
Mlynarska (2017) [37]	Hospital	none	72.7	Poland	TFI	132	Low
O’Caoimh (2017) [38]	Nursing home	none	[**84**]	Republic of Ireland	CFS	225	Low
Polidoro (2013) [39]	Hospital	none	79.3	Italy	Frailty index [59]	140	Low
Retrospective cross-sectional studies							
Annoni (2016) [2]	Hospital	≥65	84.6	Italy	Robinson criteria [**60**]	1,619	Low
Induruwa (2017) [30]	Hospital	≥75	85.3	The UK	CFS	419	Low
Lefebvre (2016) [29]	Hospital	≥80	85.9	Canada	CFS	682	Low
Prospective cohort studies							
Bo (2017) [41]	Hospital	≥65	81.6	Italy	GFI	452	Low
Doucet (2008) [42]	Hospital	>65	84.7	France	GU&G	209	Moderate
Gullón (2017) [43]	Hospital	>75	85	Spain	FRAIL scale	804	Low
Magnani (2016) [44]	Community	70–79	N/A	The USA	Health ABC battery	2,753	Low
Nguyen (2016) [32]	Hospital	≥65	84.7	Australia	Reported EFS	302	Low
Nguyen (2016) [45]	Hospital	≥65	84.7	Australia	Reported EFS	302	Low
Perera (2009) [3]	Hospital	≥70	82.7	Australia	Modified EFS	207	Low
Retrospective cohort study							
Pilotto (2016) [46]	Community, previous hospitalisation	≥65	84.4	Italy	MPI	1,287	Low

Abbreviations: EFS, Edmonton Frail Scale; GFI, Groningen frailty indicator; GU&G, get-up-and-go test; MPI, multidimensional prognostic index; MPI-SVaMA, MPI based on standardised multidimensional assessment schedule for adults and aged persons; NR, not reported; TFI, Tilburg Frailty Index. Further detail in Supplementary Table S2.

### Risk of bias within studies

Overall, the included studies were moderate to low risk of bias (Supplementary Table S3). The six studies included in the meta-analysis were judged at low risk of bias overall, with risk identified in two studies regarding ascertainment of outcome [3] and follow-up duration [3, 32]. However, these did not relate to the specific meta-analysis question of OAC and frailty associations.

### Participant characteristics

Amongst patients with AF the mean age was 83.3 years (reported in 16 studies [2, 3, 29–32, 35–37, 39, 42, 43, 45–47]), range 58–101 years (6 studies [30, 32, 39, 42, 43, 45]) and 48.2% female (18 studies [2, 3, 29–33, 35–43, 45, 46]). Excluding a large registry of outpatients [40], 56.8% of participants were female.

Eight studies also included patients without AF [2, 34–36, 38, 39, 44, 47]. The mean age of the whole cohort (those with AF and those without) was 68.5 years (reported in six studies [2, 35, 36, 39, 44, 47]), range 56–96 (two studies [35, 39]). 50.3% were female (seven studies [2, 35, 36, 38, 39, 44, 47], Supplementary Table S2).

### Assessment of frailty

Of the thirteen measures of frailty used, the timed-up-and-go test [**48**], clinical frailty scale [**49**] and Edmonton frail scale [**50**] were most common (three studies each, Table 1).

### Prevalence of AF

AF prevalence was reported in six studies, but not stratified by frailty status [2, 33–36, 38]. It varied by setting from 3% in community-dwellers [33, 35], to 38% in nursing home residents [38]. In three studies of older patients admitted acutely to hospital, AF was identified in 14% [34], 17% [36] and 24% [2] (Supplementary Table S2).

### AF and frailty

Sixteen studies reported the prevalence of frailty in patients with AF [2, 3, 29–32, 34, 36–41, 43, 45, 46]. This varied between populations, affecting 6% in a registry of outpatients aged ≥18 [40], and 100% in a nursing home population (Supplementary Table S4) [38]. In older people admitted to hospital, AF was strongly associated with being frail (adjusted OR 4.09, 95% CI 1.51–11.07, adjusted for age, sex, hypertension, diabetes, stroke, myocardial infarction and heart failure) [39].

Hung *et al.* found that whilst there was no difference in frailty between those admitted to a geriatric unit with AF and without, AF was an independent risk factor for falls (adjusted OR 1.98 [95%CI 1.08–3.63], adjusted for benzodiazepine use, paroxysmal subgroup of AF, hypertension, polypharmacy and age) [36]. However, the tendency to fall may have increased AF case-detection through use of ambulatory electrocardiography. Magnani *et al.* showed that age-related decline in physical performance in community-dwellers was accelerated by approximately ~4 years for those with AF compared with those without [44].

### AF, frailty and anticoagulation

#### Hospital cohorts

**Figure 2.**
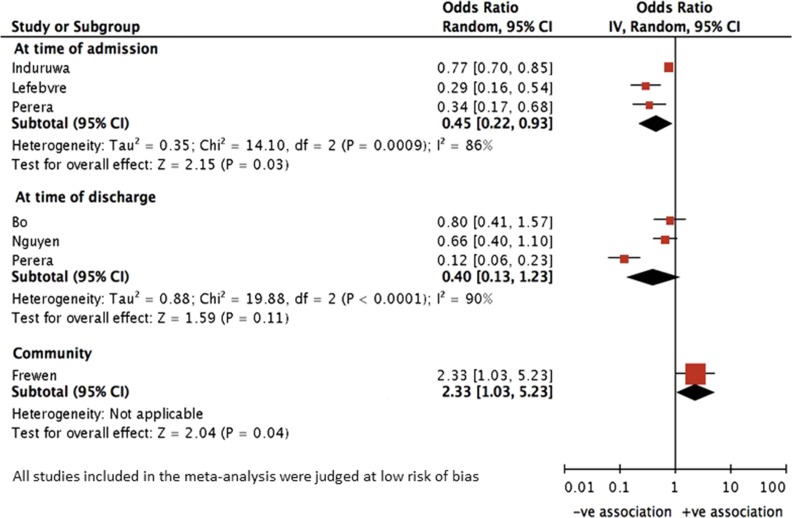
Forest plot to show association between frailty and anticoagulation status at admission, at discharge and in the community

Eight studies were in a hospitalised population with AF (Table 2) [3, 29–32, 34, 41, 42]. Five were methodologically similar, reported adjusted OR for the association between frailty and OAC, and were included in the meta-analysis (Figure 2) [3, 29–32]. Two studies reported OR at admission [29, 30], and two at discharge [31, 32]. One study reported both [3].

**Table 2.** Studies reporting the association between frailty and anticoagulation status

Study	Association: frailty and OAC prescription	Time of assessment	*n*	Unadjusted OR (95% CI)	Adjusted OR (95% CI)
Lefebvre (2016) [29]	Less use	Hospital admission	682	0.45 (0.31–0.65)	0.29 (0.16–0.54)
Induruwa (2017) [30]	Less use	Hospital admission	419	NR	0.77 (0.70–0.85)
Perera (2009) [3]	Less use	Hospital admission	220	NR	0.34 (0.17–0.68)
		Hospital discharge	220	NR	0.12 (0.06–0.23)
Denoël (2014) [34]	No difference	Hospital admission	142	OR 1.12 (0.50–2.96)	NR
Bo (2015) [31]	No difference	Hospital discharge	430	NR	0.80 (0.41–1.57)
Nguyen (2016) [32]	No difference	Hospital discharge	302	0.58 (0.36–0.93)	0.66 (0.40–1.10)
Doucet (2008) [42]	No difference	Hospital discharge	209	NR	NR
Frewen (2013) [33]	More use	Community sample	118	NR	2.33 (1.03–5.23)

Abbreviations: NR, not reported; OR, odds ratio. Adjustments detailed in Supplementary Table S5.

At hospital admission: meta-analysis showed that people with frailty had lower odds of OAC prescription than those without frailty (pooled adjusted OR 0.45 [95%CI 0.22–0.93]) [3, 29, 30]. One study reported an unadjusted OR, and was not included in the meta-analysis. This showed no association between OAC prescription and frailty (unadjusted OR 1.12 [0.50–2.96]) [34]. The later was a small study using a brief screening tool with limited predictive validity (Identifying Seniors at Risk) [**51**].

At hospital discharge: meta-analysis showed that frailty had no statistically significant association with OAC prescription (pooled adjusted OR 0.40 [95% CI 0.13−1.23]) [3, 31, 32]. One study used propensity score analysis and whilst it was not included in the meta-analysis, it also found no association between frailty and OAC prescription after matching [41].

#### Community cohorts

In contrast to the hospital cohorts, a study using a nationally representative community sample found that people with frailty had an increased odds of OAC prescription compared with people without frailty (adjusted OR 2.33 [95%CI 1.03–5.23], adjusted for age, sex and education) [47].

In a study of nursing home residents with AF and frailty, 70% of participants were eligible for OAC according to a bespoke risk based decision-support aid incorporating stroke and bleeding risk [38]. However, just 17% were prescribed OAC. A separate study found that advanced age, very short life expectancy, difficult/impossible management of therapy, fear of bleeding and harm greater than benefit were commonly reported reasons for not prescribing OAC in older patients [31].

#### DOAC use

Across five studies, DOAC was prescribed in between 5.4% and 20.6% of those anticoagulated [29–32, 43]. This was stratified by frailty status in one study, but it only included 11 patients on DOAC [30].

### Age, co-morbidity and anticoagulation

Six studies reported the association between increasing age and OAC prescription [29–32, 42, 47], five of which adjusted for other factors (Supplementary Table S5) [29–32, 47]. Increased age was independently associated with reduced OAC prescription in four studies (adjusted OR range 0.71 [0.59–0.84]–0.98 [0.97–0.98]) [29–32], but not in the fifth (adjusted OR 1.02 [0.97–1.07]) [47]. Finally, a study published in 2008 showed patients prescribed antiplatelet medications instead of OAC tended to be older (mean 86.5 vs. 82.9 years, *P* < 0.01) [42].

Two studies reported the association between Charlson co-morbidity score and OAC prescription. One showed that an increased adjusted score was independently associated with not being prescribed OAC [31]. The second showed no statistically significant difference in score between those prescribed OAC and those that were not [34].

### Anticoagulation and outcomes

One study noted a greater incidence of cardio-embolic stroke among individuals with frailty compared with those without frailty (12.3% vs. 3.9%, *P* < 0.05). However, the incident cases of stroke were not stratified by OAC prescription due to a small number of events [3]. Patients with AF and frailty also had a higher 6-month mortality compared with those with AF without frailty (unadjusted RR 2.8 [95%CI 1.2–6.5]) [3]. Nguyen *et al.* showed no difference in stroke or major bleeding by frailty status in patients with AF, which the authors suggest may be related to careful patient selection and OAC management [32].

Doucet *et al.* found no difference in clinical outcomes (stroke, death, major bleeding) at 3 months between patients with AF who were prescribed OAC compared with an antiplatelet [42]. The prevalence of falls post-discharge was higher in the aspirin compared with the OAC group (18.6% vs. 7.5%, *P* < 0.02) despite similar pre-admission falls history. This may suggest that clinicians were aware of an increased falls risk in these individuals that was not captured by the study. Physicians tended to overestimate the risk of bleeding, and underestimate the risk of thrombosis compared with objective scores.

### Frailty and mortality in AF

Three studies report the association between frailty and mortality in patients with AF. However, the different representations of risk and durations of follow-up did not allow pooling for meta-analysis. Perera *et al.* identified increased mortality in patients with AF and frailty compared with patients with AF but not frailty (unadjusted RR 2.8 [95%CI 1.2–6.5]) [3]. Nguyen *et al.* report increased 6-month mortality associated with frailty, (adjusted HR 2.33 [95%CI 1.31–4.14], adjusted for age, gender, co-morbidity, CHAD_2_DS_2_-VASc, HAS-BLED, delirium, OAC, digoxin or psychotropic medication) and that length of stay was 3.1 days longer in individuals with frailty compared with those without [45]. During a mean follow-up period of 301 days, Bo *et al.* found that in patients with AF, frailty was associated with an increased risk of mortality compared with non-frail patients (adjusted OR 2.77 [95% CI 1.44–5.33], adjusted for OAC, ADL dependence, serum albumin and readmission) [41]. A further study found that functional status, but not frailty (FRAIL scale), was independently associated with inpatient mortality [43].

## Discussion

This systematic review included 20 research articles published between 2013 and 2017. Six studies were included in a meta-analysis of the association between frailty status and OAC prescription in patients with AF. At hospital admission, frailty was associated with decreased OAC prescription, but there was no statistically significant association at discharge. A community-based study found that frailty was associated with increased OAC prescription.

We report evidence that in patients with AF, frailty is associated with increased stroke incidence [3], medium-term mortality [3, 45], symptom severity [37] and length of hospital stay [45]. One study showed frailty was not associated with stroke or major bleeding [32]. Having AF was associated with a greater chance of being frail [39], having falls [36] and physical performance decline [44] compared with people without AF, suggesting that AF itself may be a marker of frailty. There was a lack of data on clinical outcomes stratified by both frailty and OAC status [3, 32, 42].

The different association between frailty and OAC prescription among hospital and community cohorts was striking. The findings at hospital admission are reflective of prescribing patterns in the community, albeit in a subgroup who have been hospitalised, with potential for different characteristics. The absence of a statistically significant association between OAC prescription and frailty status at discharge may be because hospitalisation allowed more complete case ascertainment and prescription of therapy. However, survivorship bias is also a potential factor, whereby fitter patients are more likely to survive to discharge. Furthermore, hospitalisation in the context of frailty is a potential marker of nearing end of life, so de-prescribing decisions could be influenced accordingly [52].

In a community study with a relatively young population and low AF prevalence, frailty was associated with an increased OAC prescription rate [47]. In contrast, in a nursing home population with a relatively high prevalence, just 25% of the eligible population were prescribed OAC [38]. Competing risks are likely to be influencing prescribing behaviour in this vulnerable population.

There are concerns that clinical guidelines tend to relate to single-organ pathology [10, **13**], and the trial evidence on which they are based frequently excludes people with frailty, including of DOACs [**17–20**]**. **Furthermore, CHA_2_DS_2_-VASc has not been validated for use in the oldest old or people with frailty [**53**]. In the absence of trial evidence, observational data can offer insights into current practice and patient outcomes. However, this review identified a lack of research in a community setting using validated frailty measures, despite growing evidence that a greater mortality risk is carried by measures of biological than chronological age [9, 54]. There is therefore a limited evidence base to guide management in this high-risk population in whom bleeding complications may be more common and more problematic than in the general population [**16, 55**]. A risk-treatment paradox exists, whereby those at the highest risk of stroke are not more likely to receive anticoagulation [**6, 56**]. Whether frailty should influence OAC prescribing, including through incorporation into AF decision-support tools, is currently unknown.

## Strengths of the review

To our knowledge, this is the first systematic review to summarise current evidence for the management of AF in older people with frailty. We have used a robust search strategy, risk of bias assessment and methods pre-specified in a published protocol. We were able to present pooled adjusted estimates of the association between OAC prescription and frailty, and included data on DOAC use, reflecting recent trends. However, the small proportion of patients that were taking DOAC in the included studies despite its increasing role reinforces the need for contemporary research [57].

## Limitations of the review

A range of frailty measures was used and frailty was dichotomised as in the source study. This may introduce additional heterogeneity in the meta-analysis. Whilst we have reported OAC prescription at different time points, this was without access to individual patient data, so we cannot exclude misclassification error. Frailty was often diagnosed in an acute hospital setting, although guidance suggests frailty assessment is best performed in the community [58]. Most studies excluded patients with cognitive or major sensory impairment due to the necessity for informed consent, and so may not be representative of the overall frail population. Some studies required participants to complete a physical task, which may exclude those with advanced frailty. There was variation in the choice of confounders in the adjusted estimates included in our meta-analysis. We have reported adjusted and unadjusted estimates where available, with similar direction of associations.

As with any meta-analysis of observational data there are risks of confounding by indication and other systemic biases that are incompletely accounted for. Further observational data in a community setting with complementary qualitative work would contribute to our understanding of current practice, but with susceptibility to bias. A randomised trial may ultimately be needed to help quantify efficacy and safety endpoints in a frail population.

## Conclusion

At hospital admission frailty was associated with decreased OAC prescription. However, there was no statistically significant association at the time of discharge. A single study in a community setting showed that frailty was associated with increased OAC prescription. There is evidence that in patients with AF, frailty is associated with increased stroke incidence, mortality, symptom severity, and length of hospital stay.

Although anticoagulation is largely initiated and managed in primary care, there is a lack of evidence to guide optimal care in this setting for patients with AF and frailty. This may in part explain a gap between current guidelines and clinical practice in management of these patients, particularly in relation to OAC prescription.

## Supplementary Material

Supplementary Data

## Notes

**Acknowledgements:** The authors are grateful to Deidre Andre, Research Librarian at the University of Leeds for assistance with the design of the search strategies.

**Declaration of Conflict of Interest:** None.

**Funding:** This work was supported by the Dunhill Medical Trust (RTF107/0117) research training fellowship to OT; Wellcome Trust (206470/Z/17/Z) Sir Henry Wellcome Postdoctoral Fellowship to MH. AC is part-funded by the National Institute for Health Research Collaboration for Leadership in Applied Health Research and Care, Yorkshire & Humber. The funders had no role in the design, execution, analysis or interpretation of the data or writing of the study.
