# Supplementary material for: Management of atrial fibrillation for older people with frailty: a systematic review and meta-analysis
Source: Age Ageing. 2018 Nov 15;48(2):196–203. doi: 10.1093/ageing/afy180 (PMC6424377; doi:10.1093/ageing/afy180)
Supplement: Supplementary Data [file afy180_aa-18-0478-file002.docx]

**Management of atrial fibrillation for older people with frailty:
a systematic review and meta-analysis**

**Supplementary Data**

| Search strategy for Ovid Medline | 2 |
| --- | --- |
| Table 1: Summary of included studies | 3 |
| Table 2: Summary of participant characteristics in included studies | 7 |
| Table 3: Risk of bias assessment | 9 |
| Table 4: Reported prevalence and definitions of frailty in included studies | 11 |
| Table 5: Studies reporting association between frailty and anticoagulation status | 12 |
| List of adjustments used to evaluate factors associated with receipt of evidence-based therapy among patients with AF, Hess et al [40] | 14 |
| Complete reference list | 15 |

**Search strategy for Ovid Medline. Rows combined with 'OR', columns combined with 'AND'**

We searched CINAHL, Cochrane Trials and Cochrane Database of systematic reviews, Embase, Ovid Medline, Medline In-Process & Other Non-Indexed Citations, and Web of Science from inception of each until October 2017.

| atrial fibrillation/ | frail elderly/ |
| --- | --- |
| atrial fibrillation*.tw. | (frail* or sarcop?eni* or prefrailty).tw. |
| auricular fibrillation*.tw. | sarcopenia/ |
| atrium fibrillation*.tw. | Geriatric Assessment/ |
| catheter ablation/ | "comprehensive geriatric assessment".tw. |
| atrial ablation*.tw. | (multimorbid* or multi-morbid*).tw. |
| (electric* adj2 ablation*).tw. | (multidisease? or multi-disease? or (multiple adj (ill* or disease? or condition? or syndrom* or disorder?))).tw. |
| catheter ablation*.tw. | geriatric syndrom*.tw. |
| (radiofrequency adj2 ablation*).tw. | cumulative deficit*.tw. |
| pulmonary vein isolation*.tw. | Phenotype model*.tw. |
| exp Anti-Arrhythmia Agents/ | ((Edmonton or Fried) adj5 (index* or indicator* or score* or scale* or tool* or test* or model* or phenotype* or criteri* or marker* or method* or instrument* or assess* or exam* or evaluat* or measure* or screen* or diagnos* or detect* or identif*)).tw. |
| Atrial Flutter/ | (Gait speed* or walking speed* or grip strength*).tw. |
| atrial flutter.tw. | exp hand strength/ |
| atrium flutter.tw. | ("Timed up and go test*" or tugt or gug or "get up and go").tw. |
| Tachycardia, Ectopic Atrial/ | frail elderly/ |
| ((atrial or atrium or auricular) adj2 (tachycardia* or tachyarrhythmia*)).tw. | (frail* or sarcop?eni* or prefrailty).tw. |
| antiarrhythmi*.tw. | sarcopenia/ |
| anti-arrhythmi*.tw. | Geriatric Assessment/ |
| exp Anticoagulants/ | "comprehensive geriatric assessment".tw. |
| anticoagulant*.tw. | (multimorbid* or multi-morbid*).tw. |
| antithrombotic*.tw. | (multidisease? or multi-disease? or (multiple adj (ill* or disease? or condition? or syndrom* or disorder?))).tw. |

Symbols: * = Truncation. This identifies variant endings for the stem word

? = Wildcard. This allows a different character (or no character) to identify variant spellings of words.

**Table 1: Summary of included studies**

| **Study** | **Study type** | **Study population** | **Age criteria** | **Number of centres** | **Country** | **Measure of frailty** | **n** | **Summary** |
| --- | --- | --- | --- | --- | --- | --- | --- | --- |
| Annoni 2016 [2] | Retrospective cross-sectional | Consecutive admissions to acute geriatric unit | ≥65 | 1 | Italy | Robinson criteria [60] | 1619 | AF prevalence 24.9%.86 % of those with AF were frail or pre-frail. Those with AF had more comorbidities and medications. |
| Bo 2015 [31] | Prospective cross-sectional | Admissions with AF to internal medicine | ≥65 | 3 | Italy | GFI | 513 | 78% were frail. 49% were on OAC at discharge.  Age and co-morbidities were independently associated with lack OAC; frailty was not. Common reasons for not anticoagulating: advanced age, life expectancy, difficult management of therapy, perceived fear of harm including bleeding. |
| Bo 2017 [41] | Prospective cohort | Discharges with AF | ≥65 | 3 | Italy | GFI | 452 | 33% of patients died within mean follow-up of 301 days.  OAC prescribed at discharge in 50%, and was associated with decreased mortality and ischaemic stroke. After propensity matching, frailty status was not associated with OAC use. |
| Denoël 2014 [34] | Prospective cross-sectional | Consecutive admissions to ED with AF. | ≥75 | 1 | Belgium | ISAR | 995 | AF prevalence 14%. OAC was guideline-recommended for 71%, and prescribed in 61%.  OAC use not associated with CHADS_2_ score or geriatric characteristics. |
| Donoghue 2014 [35] | Prospective cross-sectional | Mobile, community-dwelling participants in the TILDA study | ≥50 | N/A | Republic of Ireland | GU&G  Gait speed | 4525 | AF prevalence 3.1% overall, 4.7% aged >70.  AF independently associated with slower TUG and usual gait speed. Adults with AF at age 70 walked 3.8 cm/s more slowly than those without. The difference increased with age, and persisted after adjustment. |
| Doucet 2008 [42] | Prospective cohort | Consecutive admissions with AF | >65 | 2 | France | GU&G | 209 | 49% discharged on OAC, the rest on aspirin. Physicians overestimated bleeding and underestimated thrombosis risks.  There was no difference in GU&G scores between the groups, or in stroke, haemorrhage or death at 3 months. |
| Frewen 2013 [47] | Prospective cross-sectional | Mobile, community-dwelling participants in the TILDA study | ≥50 | N/A | Republic of Ireland | Fried criteria | 4890 | AF prevalence 3%. 41% on OAC if CHA_2_DS_2_-VASc ≥2  OR for non-treatment with OAC associated with frailty 0.43 (95%CI 0.19-0.96). |
| Gullón 2017 [43] | Prospective cohort | Inpatients with NVAF | >75 | 64 | Spain | 5 item FRAIL scale | 804 | 50% were frail. Frailty was not independently associated with mortality, but total dependency was – OR 4.73 (2.32-9.63)  All-cause in-hospital mortality 10%. |
| Hess 2013 [40] | Prospective cohort | Outpatients in registry | ≥18 | 174 | USA | Health ABC physical performance battery | 10,096 | Frailty was significantly associated with not receiving evidence based therapy for co-morbidities, OR 0.75 (0.59-0.95). |
| Hung 2013 [36] | Prospective cross-sectional | Admissions to geriatric unit | ≥75 | 1 | Taiwan | GU&G | 401 | 71.2% of patients with AF had a history of falls.  AF was an independent risk factor for falls, OR 1.98 (1.08-3.63). |
| Induruwa 2017 [30] | Retrospective cross-sectional | General medical admissions with AF. | none | 1 | England | CFS | 419 | 51% were not on OAC.  Frailty was an independent predictor for non-use of OAC, OR 0.77 (0.70-0.85). |
| Lefebvre 2016 [29] | Retrospective cross-sectional | Admissions with AF | ≥80 | 3 | Canada | CFS | 682 | 70% on OAC, 20.6% of these with DOAC.  Compared with severely frail patients, non-frail to moderately frail had adjusted OR for OAC 3.41 (1.84-6.33).  CHADS_2_ score was positively (and HAS-BLED negatively) correlated with OAC |
| Magnani 2016 [44] | Prospective cohort | Community based cohort receiving Medicare  Two cities. | 70-79 | N/A | USA | Health ABC physical performance battery | 2753 | There was an accelerated progressive decline of physical performance in cohort participants with AF compared with those without.  AF appears to be a marker of frailty that is associated with exacerbated decline. |
| Mlynarska 2017 [37] | Prospective cross-sectional | Inpatients with AF | none | 1 | Poland | TFI | 132 | 60% were frail. Frailty was associated with a lower acceptance of AF diagnosis and a greater reported intensity of symptoms. |
| Nguyen 2016 [32] | Prospective cohort | Inpatients with AF | ≥65 | 1 | Australia | Reported EFS | 302 | 53% were frail. 51% of whole cohort on OAC, but use not independently associated with frailty. Greater use of digoxin in frail people (34.7% vs 23% p0.03), but not in other anti-arrhythmic use. No difference in bleeding or stroke by frailty. |
| Nguyen 2016 [45] | Prospective cohort | Inpatients with AF | ≥65 | 1 | Australia | Reported EFS | 302 | Adjusted HR for mortality associated with frailty 2.33 (1.31-4.14). LOS longer in the frail, 14.1 vs 11 days, p0.002.  No difference in readmissions by frailty. |
| O’Caoimh 2017 [38] | Prospective cross-sectional | Frail NH residents. | none | 4 | Republic of Ireland | CFS | 225 | AF prevalence 38%. All had CHA_2_DS_2_-VASc ≥2. 17% were anticoagulated, but a risk based decision support aid suggested that 70% should be. |
| Perera 2009 [3] | Prospective cohort | Inpatients with AF | ≥70 | 1 | Australia | Modified EFS | 207 | 63% were frail, and this was negatively associated with OAC use. Increased likelihood of death or embolic stroke with in the frail, but not stratified by OAC status. |
| Pilotto 2016 [46] | Retrospective cohort | Community-dwelling adults with previous hospitalisation for AF | ≥65 | N/A | Italy | MPI | 1287 | 44% on OAC. Tended to be younger, with better cognitive status and MPI-SVaMA.  Overall mortality reduction with warfarin regardless of MPI-SVaMA group, HR 0.6 (0.6-0.7) over mean 2 year follow-up. |
| Polidoro 2013 [39] | Prospective cross-sectional | Consecutive admissions to geriatric unit. |  | 1 | Italy | Frailty index[59] | 140 | AF was associated with frailty status, OR 4.09 (95%CI 1.51-11.07).  The authors suggest that AF could be a useful marker of frailty. |
| Abbreviations  AF: atrial fibrillation, DOAC: direct oral anticoagulant, ED: emergency department, EFS: Edmonton Frail Scale, GFI: Groningen frailty indicator, GU&G: get-up-and-go test, LOS: length of stay, MPI: multidimensional prognostic index, MPI-SVaMA: MPI based on standardized multidimensional assessment schedule for adults and aged persons, NH: nursing home, NVAF: non-valvular AF, OR: odds ratio, TFI: Tilburg Frailty Index, TILDA: the Irish longitudinal study on ageing | | | | | | | | |

**Table 2: Summary of participant characteristics in included studies**

|  |  |  | | **Participants with AF** | | | | | **Whole cohort (those with and without AF)** | | | | |
| --- | --- | --- | --- | --- | --- | --- | --- | --- | --- | --- | --- | --- | --- |
| **Study** | **Setting** | **Inclusion age** | **AF prevalence** | **n** | **Mean age**  **[median]** | **SD**  **[IQR]** | **Range** | **% female** | **n** | **Mean age**  **[median]** | **SD**  **[IQR]** | **Range** | **% female** |
| Annoni [2] | Inpatients | ≥65 | 24.3% | 403 | 84.6 | 3.2 | NR | 59.6% | 1619 | 84.1 | 6.7 | NR | 59.7% |
| Bo, 2015 [31] | Inpatients | ≥65 | - | 631 | 81.7 | 6.8 | NR | 55.6% | - | - | - | - | - |
| Bo, 2017 [41] | Inpatients | ≥65 | - | 513 | 81.6 | 6.6 | NR | 54.9% | - | - | - | - | - |
| Denoël^1^ [34] | Inpatients | ≥75 | 14% | 142 | NR | NR | NR | NR | 995 | NR | NR | NR | NR |
| Donoghue [35] | Community | ≥50 | 3.1% | 112 | 70.7 | 8.6 | NR | 21.1% | 4525 | 63.5 | 8.9 | 51-89 | 52.1% |
| Doucet [42] | Inpatient | ≥65 | - | 228 | 84.7 | 7.0 | 65-100 | 60.8% | - | - | - | - | - |
| Frewen [47] | Community | ≥50 | 3% | 118 | 63.8 | 9.8 | NR | 46% | 4890 | 63.8 | 9.8 | NR | 46% |
| Gullón [43] | Inpatient | >75 | - | 804 | 85 | 5.1 | 75-101 | 53.9% | - | - | - | - | - |
| Hess^1^ [40] | Outpatients | ≥18 | - | 10,096 | [75] | [67-82] | NR | 42.6% | - | - | - | - | - |
| Hung [36] | Inpatient | ≥75 | 16.5% | 66 | 82.6 | 0.6 | NR | 26% | 401 | 82.2 | 0.2 | NR | 24% |
| Induruwa^2^ [30] | Inpatients | ≥75 | - | 419 | 85.3 | 5.6 | 75-101 | 54.9% | - | - | - | - | - |
| Lefebvre [29] | Inpatients | ≥80 | - | 682 | 85.9 | 4.4 | NR | 60.4% | - | - | - | - | - |
| Magnani [44] | Community | 70-79 | - | N/A3 | N/A | N/A | N/A | N/A | 2753 | 73.6 | 2.9 | NR | 52% |
| Mlynarska [37] | Inpatients | None | - | 132 | 72.7 | 6.73 | NR | 44.7% | - | - | - | - | - |
| Nguyen [32] | Inpatients | ≥65 | - | 302 | 84.7 | 7.1 | 65-100 | 50% | - | - | - | - | - |
| Nguyen [45] | Inpatients | ≥65 | - | 302 | 84.7 | 7.1 | 65-100 | 50% | - | - | - | - | - |
| O’Caoimh [38] | Nursing homes | None | 38% | 86 | [84] | [78-89] | NR | 37% | 225 | [85] | [77-89] | NR | 60% |
| Perera [3] | Inpatients | ≥70 | - | 220 | 82.7 | 6.3 | NR | 54 | - | - | - | - | - |
| Pilotto [46] | Previous hospitalisation | ≥65 | - | 1827 | 84.4 | 7.1 | NR | 64.3% | - | - | - | - | - |
| Polidoro [39] | Inpatients | None | - | 70 | 79.3 | 7.5 | 58-96 | 59% | 140 | 79.2 | 7.4 | 56-96 | 59% |
| Abbreviations: NR: not reported, N/A: not applicable  1 Author contacted for further information, but no response; 2 Author kindly provided additional information for completeness; 3 Patients with prevalent AF were excluded. Data reported are from baseline visit. The study reports incident AF. | | | | | | | | | | | | | |

**Table 3:** Risk of bias assessment

The Newcastle-Ottawa Scale was used for quality assessment, adapted for cross-sectional studies.[25, 26] A maximum of one star is awarded for each heading under selection and outcome, and two stars under comparability. The total possible is seven stars for cross-sectional studies. Good: ≥5; moderate: 3-4; poor ≤2. For cohort studies, the total possible is nine stars. Good: ≥7; moderate: 5-6; poor ≤4

| 3A: Cross-sectional studies | | | | | | | |
| --- | --- | --- | --- | --- | --- | --- | --- |
|  | **Selection** | | | **Comparability** | **Outcome** | | **Total** |
|  | Representative of exposed cohort | Selection of non-exposed cohort | Ascertainment of exposure | Controls and adjusted | Ascertainment of outcome | Statistical test |  |
| Annoni, 2016 [2] | 1 | 1 | 1 | 1 | 0 | 1 | 5 |
| Bo, 2015 [31] | 1 | 1 | 1 | 2 | 1 | 1 | 7 |
| Denoël, 2014 [34] | 1 | 1 | 1 | 2 | 0 | 1 | 6 |
| Donoghue, 2014 [35] | 1 | 1 | 1 | 2 | 1 | 1 | 7 |
| Frewen, 2013 [47] | 1 | 1 | 1 | 2 | 0 | 1 | 6 |
| Hess, 2013 [40] | 1 | 1 | 1 | 2 | 1 | 1 | 7 |
| Hung, 2013 [36] | 1 | 1 | 1 | 2 | 1 | 1 | 7 |
| Induruwa, 2017 [30] | 1 | 1 | 1 | 2 | 1 | 1 | 7 |
| Lefebvre, 2016 [29] | 1 | 1 | 1 | 2 | 1 | 1 | 7 |
| Mlynarska, 2017 [37] | 1 | 1 | 1 | 1 | 1 | 1 | 6 |
| O’Caoimh, 2017 [38] | 1 | 1 | 1 | 1 | 0 | 1 | 5 |
| Polidoro, 2013 [39] | 1 | 1 | 1 | 1 | 1 | 1 | 6 |

| 3B: Cohort studies | | | | | | | | | |
| --- | --- | --- | --- | --- | --- | --- | --- | --- | --- |
|  | **Selection** | | | | **Comparability** | **Outcome** | | | **Total** |
|  | Representative of exposed cohort | Selection of non-exposed cohort | Ascertainment of exposure | Outcome not present at start | Controls and adjusted | Ascertainment of outcome | Was follow up long enough | Adequate follow-up |  |
| Bo, 2017 [41] | 1 | 1 | 1 | 1 | 2 | 1 | 0 | 0 | 7 |
| Doucet, 2008 [42] | 1 | 1 | 1 | 1 | 1 | 1 | 0 | 0 | 6 |
| Gullón, 2017 [43] | 1 | 0 | 1 | 1 | 2 | 1 | 1 | 1 | 8 |
| Magnani., 2016 [44] | 1 | 1 | 1 | 1 | 2 | 1 | 1 | 0 | 8 |
| Nguyen, 2016 [32] | 1 | 1 | 1 | 1 | 2 | 1 | 0 | 0 | 7 |
| Nguyen, 2016 [45] | 1 | 1 | 1 | 1 | 2 | 1 | 0 | 0 | 7 |
| Perera, 2009 [3] | 1 | 1 | 1 | 1 | 2 | 0 | 0 | 1 | 7 |
| Pilotto, 2016 [46] | 1 | 0 | 1 | 1 | 2 | 1 | 1 | 1 | 8 |

**Table 4:** Reported prevalence and definitions of frailty in included studies

| **Study** | **Mean age [median], patients with AF** | **Frailty definition** | | **Frailty prevalence** | |
| --- | --- | --- | --- | --- | --- |
|  |  | **Measure** | **Cut-point** | **Whole cohort** | **Patients with AF** |
| Annoni [2] | 84.6 | Robinson criteria [60] | ≥4 | NR | 57.3% |
| Bo [31] | 81.7 | GFI | ≥4 | - | 77.5% |
| Bo [41] | 81.6 | GFI | ≥4 | - | 75.4% |
| Denoël [34] | NR | ISAR | ≥2 | NR | 84% |
| Donoghue [35] | 70.7 | GU&G  Gait speed | Comparison was made between groups with AF and without AF, no threshold was used | | |
| Doucet [42] | 84.7 | GU&G | Comparison was made between those prescribed OAC and those that weren’t. | | |
| Frewen [47] | 63.8 | Fried criteria | ≥1 | NR | NR |
| Gullón [43] | 85 | FRAIL scale | ≥3 | - | 50.3% |
| Hess [40] | [75] | Fried criteria | ≥3 | - | 6.0% |
| Hung [36] | 82.6 | GU&G | >10 seconds | 87% | 83% |
| Induruwa [30] | 85.3 | CFS | 5-8 | - | 67.3% |
| Lefebvre [29] | 85.9 | CFS | ≥7 | - | 25.4% |
| Magnani [44] | N/A | Health ABC PPB | Scores were compared over time for the same individuals, and the effect of developing AF estimated | | |
| Mlynarska [37] | 72.7 | TFI | ≥5 | - | 60% |
| Nguyen [32] | 84.7 | Reported EFS | ≥8 | - | 53.3% |
| Nguyen [45] | 84.7 | Reported EFS | ≥8 | - | 53.3% |
| O’Caoimh [38] | [84] | CFS | ≥5  *≥7** | -  - | 100%  *85.8%* |
| Perera [3] | 82.7 | Modified EFS | ≥8 | - | 64% |
| Pilotto [46] | 84.4 | MPI | ≥2 | - | 61.4% |
| Polidoro [39] | 79.3 | Frailty index[59] | 0.25 | 77.9% | 88.6% |
| * Threshold of 5 used by the authors. Results for a threshold of 7 also reported in this table for comparison purposes. Abbreviations: CFS: clinical frail scale, EFS: Edmonton frail scale, GFI: Groningen frailty indicator, GU&G: get up and go, ISAR: Identification of seniors at risk, MPI: multidimensional prognostic index, N/A: not-applicable, NR: not reported, PPB: physical performance battery, OAC: oral anticoagulant, TFI: Tilburg frailty indicator | | | | | |

**Table 5: Studies reporting association between frailty and anticoagulation status**

| **Study** | **Association: frailty and OAC use** | **Setting** | **n=** | **Estimate**  **(95% CI)** | **Adjustments** |
| --- | --- | --- | --- | --- | --- |
| Lefebvre, 2016 [29] | Less use | Hospital  OAC as inpatient | 682 | OR 0.29  (0.16-0.54) | Falls history, CHADS_2_ score, bleeding risk, age, length of hospital stay, use of antiplatelet agents or medication that increases bleeding risk) |
| Induruwa, 2017 [30] | Less use | Hospital  OAC at time of admission | 419 | OR 0.77  (0.70–0.85) | Age, sex, and the components of CHA_2_DS_2_-VASc and HAS-BLED |
| Perera, 2009 [3] | Less use | Hospital  OAC at time of admission | 220 | OR 0.34  (0.17-0.68) | Age, CCS, Gender, herbal medications, admission ward, nutritional status number of medications, MMSE, Katz Daily Living Score, alcohol use, excessive falls risk, anaemia, previous adverse reaction to warfarin, previous adverse reaction to aspirin, previous haemorrhagic stroke, malignancy, reduced platelet count, previous major bleeding episode, uncontrolled hypertension, age > 75 years, diabetes mellitus, hypertension congestive heart failure, prior stroke |
|  |  | At time of discharge | 220 | OR 0.12  (0.06-0.23) |  |
| Denoël, 2014 [34] | No difference | Hospital  OAC at time of admission | 142 | OR 1.12  (0.50-2.96) | Unadjusted |
| Bo, 2015 [31] | No difference | Hospital  OAC at time of discharge | 430 | OR 0.80  (0.41–1.57) | Age, AF subtype, CHA_2_DS_2_-VASc, HAS-BLED, CCI, ADL dependence, cognitive impairment, depression, malnutrition, discharge to a facility |
| Bo, 2017 [41] | No difference | Hospital  OAC at time of discharge | 452 | N/A | Propensity score analysis using a 1:1 nearest-neighbour-matching algorithm |
| Nguyen, 2016 [32] | No difference | Hospital  OAC at time of discharge | 302 | OR 0.66  (0.40–1.10) | Age, history of bleeding/ predisposition to bleeding and abnormal renal function, congestive heart failure |
| Doucet, 2008 [42] | No difference | Hospital  OAC at time of discharge | 209 | N/A | Simple comparison between groups, no adjustment |
| Frewen, 2013 [33] | More use | Nationally representative community sample from the TILDA study. | 118 | OR 2.33  (1.03-5.23) | Age, sex and education |
| Abbreviations: ADL: Activities of Daily Living, CCS: Charlson Comorbidity Score, CFS: Clinical Frail Scale, CHADS_2_: Congestive Heart Failure, Hypertension, Age, Diabetes, Stroke/Transient Ischemic Attack. CHA_2_DS_2_-VASc: Congestive heart failure, Hypertension, Diabetes, Stroke/Transient Ischaemic Attack, Vascular disease. EFS: Edmonton Frail Scale, GFI: Groningen Frailty indicator, HAS-BLED: Liver abnormalities, Renal abnormalities, Prior bleeding or disposition, Labile INR, Alcohol, and Medication with bleeding risk. ISAR: Identification of Seniors at Risk, OAC: Oral Anticoagulant, OR: odds ratio, TILDA: The Irish longitudinal study on ageing. | | | | | |

**List of adjustments used to evaluate factors associated with receipt of evidence-based therapy among patients with AF, Hess et al [40]**

Variables in the model included: age, sex, race, insurance status, educational status, body mass index, heart rate, thyroid disease, obstructive sleep apnoea, cognitive impairment, liver disease, alcohol abuse, cancer, osteoporosis, hip fracture, gastrointestinal bleed, dialysis dependence, anaemia, frailty, chronic obstructive pulmonary disease, drug abuse, family history of atrial fibrillation, sinus node dysfunction or sick sinus syndrome, estimated glomerular filtration rate, current antiarrhythmic drug use, current smoking, catheter ablation of atrial fibrillation, prior stroke or transient ischemic attack, renal insufficiency, past warfarin use, current warfarin use, contraindications to oral anticoagulant therapy, functional status, and provider specialty.

# Complete reference list

1. Zoni-Berisso M, Lercari F, Carazza T, Domenicucci S. Epidemiology of atrial fibrillation: European perspective. Clinical Epidemiology. 2014 06/16;6:213-20.

2. Annoni G, Mazzola P. Real-world characteristics of hospitalized frail elderly patients with atrial fibrillation: can we improve the current prescription of anticoagulants? Journal of Geriatric Cardiology. 2016 2016;13(3):226-32.

3. Perera V, Bajorek BV, Matthews S, Hilmer SN. The impact of frailty on the utilisation of antithrombotic therapy in older patients with atrial fibrillation. Age and Ageing. 2009 Mar;38(2):156-62.

4. Wolff A, Shantsila E, Lip GYH, Lane DA. Impact of advanced age on management and prognosis in atrial fibrillation: insights from a population-based study in general practice. Age and Ageing. 2015;44(5):874-8.

5. Cowan C, Healicon R, Robson I, Long WR, Barrett J, Fay M, et al. The use of anticoagulants in the management of atrial fibrillation among general practices in England. Heart. 2013;99(16):1166-72.

6. Sandhu RK, Bakal JA, Ezekowitz JA, McAlister FA. Risk stratification schemes, anticoagulation use and outcomes: the risk–treatment paradox in patients with newly diagnosed non-valvular atrial fibrillation. Heart. 2011;97(24):2046-50.

7. Monette J, Gurwitz JH, Rochon P, Avorn J. Physicians' knowledge and attitudes regarding the use of warfarin in frail elderly patients with atrial fibrillation. Journal of the American Geriatrics Society. 1996 Sep;44(9):P45-P.

8. Monette J, Gurwitz JH, Rochon P, Glynn RJ, Avorn J. Warfarin in the frail elderly with atrial fibrillation: Results of a physician survey. Clinical Pharmacology & Therapeutics. 1996 Feb;59(2):PII56-PII.

9. Clegg A, Young J, Iliffe S, Rikkert MO, Rockwood K. Frailty in elderly people. Lancet. 2013 Mar 2;381(9868):752-62.

10. National Institute for Health and Care Excellence. Multimorbidity: clinical assessment and management. NICE guideline NG56. 2016.

11. Gale CR, Cooper C, Aihie Sayer A. Prevalence of frailty and disability: findings from the English Longitudinal Study of Ageing. Age and Ageing. 2015;44(1):162-5.

12. Rich MW, Chyun DA, Skolnick AH, Alexander KP, Forman DE, Kitzman DW, et al. Knowledge Gaps in Cardiovascular Care of Older Adults: A Scientific Statement from the American Heart Association, American College of Cardiology, and American Geriatrics Society: Executive Summary. Journal of the American Geriatrics Society. 2016 Nov;64(11):2185-92.

13. Walker DM, Gale CP, Lip G, Martin-Sanchez FJ, McIntyre HF, Mueller C, et al. Frailty and the management of patients with acute cardiovascular disease: A position paper from the Acute Cardiovascular Care Association. European heart journal Acute cardiovascular care. 2018 Mar;7(2):176-93.

14. Morley JE, Vellas B, van Kan GA, Anker SD, Bauer JM, Bernabei R, et al. Frailty Consensus: A Call to Action. Journal of the American Medical Directors Association. 2013;14(6):392-7.

15. Pisters R, Lane DA, Nieuwlaat R, de Vos CB, Crijns HJ, Lip GY. A novel user-friendly score (HAS-BLED) to assess 1-year risk of major bleeding in patients with atrial fibrillation: the Euro Heart Survey. Chest. 2010 Nov;138(5):1093-100.

16. Bahat G, Ilhan B, Karan MA. HAS-BLED score: Limitations due to underestimation of bleeding risk in the elderly. Nobel Medicus. 2015 01 May;11(2):101-2.

17. Patel MR, Mahaffey KW, Garg J, Pan G, Singer DE, Hacke W, et al. Rivaroxaban versus Warfarin in Nonvalvular Atrial Fibrillation. New England Journal of Medicine. 2011;365(10):883-91.

18. Granger CB, Alexander JH, McMurray JJV, Lopes RD, Hylek EM, Hanna M, et al. Apixaban versus Warfarin in Patients with Atrial Fibrillation. New England Journal of Medicine. 2011;365(11):981-92.

19. Giugliano RP, Ruff CT, Braunwald E, Murphy SA, Wiviott SD, Halperin JL, et al. Edoxaban versus Warfarin in Patients with Atrial Fibrillation. New England Journal of Medicine. 2013;369(22):2093-104.

20. Connolly SJ, Ezekowitz MD, Yusuf S, Eikelboom J, Oldgren J, Parekh A, et al. Dabigatran versus Warfarin in Patients with Atrial Fibrillation. New England Journal of Medicine. 2009;361(12):1139-51.

21. Stroup DF, Berlin JA, Morton SC, Olkin I, Williamson GD, Rennie D, et al. Meta-analysis of observational studies in epidemiology: a proposal for reporting. Meta-analysis Of Observational Studies in Epidemiology (MOOSE) group. Jama. 2000 Apr 19;283(15):2008-12.

22. Liberati A, Altman DG, Tetzlaff J, Mulrow C, Gøtzsche PC, Ioannidis JPA, et al. The PRISMA statement for reporting systematic reviews and meta-analyses of studies that evaluate healthcare interventions: explanation and elaboration. BMJ. 2009;339.

23. Wilkinson C, Todd O, Clegg A, Gale C, Hall M. How is frailty associated with the prevalence and clinical outcomes of atrial fibrillation? PROSPERO. CRD42018092951. 2018; Available from: <http://www.crd.york.ac.uk/PROSPERO/display_record.php?ID=CRD42018092951>.

24. Veritas Health Innovation. Covidence systematic review software. Melbourne, Australia; Available from: [www.covidence.org](http://www.covidence.org).

25. Wells G, Shea B, O'Connell D, Peterson J, Welch V, Losos M, et al. The Newcastle-Ottawa Scale (NOS) for assessing the quality of nonrandomised studies in meta-analyses. Canada2009 [29/11/2017]; Available from: <http://www.ohri.ca/programs/clinical_epidemiology/oxford.asp>.

26. Higgins JPT, Altman DG, Sterne JAC. Assessing risk of bias in included studies. In: Higgins J, Green S, editors. Cochrane Handbook for Systematic Reviews of Interventions Version 510. London: Cochrane Collaboration; 2011.

27. Ong T, Kantachuvesiri P, Sahota O, Gladman JRF. Characteristics and outcomes of hospitalised patients with vertebral fragility fractures: a systematic review. Age and Ageing. 2018;47(1):17-25.

28. The Cochrane Collaboration. Review Manageer (RevMan) 5.3. Copenhagen: The Nordic Cochrane Centre2014.

29. Lefebvre M-CD, St-Onge M, Glazer-Cavanagh M, Bell L, Nguyen JNK, Nguyen PV-Q, et al. The Effect of Bleeding Risk and Frailty Status on Anticoagulation Patterns in Octogenarians With Atrial Fibrillation: The FRAIL-AF Study. Canadian Journal of Cardiology. 2016 Feb;32(2):169-76.

30. Induruwa I, Evans NR, Aziz A, Reddy S, Khadjooi K, Romero-Ortuno R. Clinical frailty is independently associated with non-prescription of anticoagulants in older patients with atrial fibrillation. Geriatrics & gerontology international. 2017 2017-Apr-18.

31. Bo M, Li Puma F, Badinella Martini M, Falcone Y, Iacovino M, Grisoglio E, et al. Health status, geriatric syndromes and prescription of oral anticoagulant therapy in elderly medical in-patients with atrial fibrillation: a prospective observational study. International Journal of Cardiology. [Letter]. 2015;187:123-5.

32. Nguyen TN, Cumming RG, Hilmer SN. Atrial fibrillation in older inpatients: are there any differences in clinical characteristics and pharmacological treatment between the frail and the non-frail? Internal Medicine Journal. 2016 Jan;46(1):86-95.

33. Frewen J, Finucane C, Cronin H, Rice C, Kearney PM, Harbison J, et al. Factors that influence awareness and treatment of atrial fibrillation in older adults. Qjm-an International Journal of Medicine. 2013 May;106(5):415-24.

34. Denoel P, Vanderstraeten J, Mols P, Pepersack T. Could some geriatric characteristics hinder the prescription of anticoagulants in atrial fibrillation in the elderly? Journal of aging research. 2014 2014;2014:693740-.

35. Donoghue OA, Jansen S, Dooley C, De Rooij S, Van Der Velde N, Kenny RA. Atrial Fibrillation Is Associated With Impaired Mobility in Community-Dwelling Older Adults. Journal of the American Medical Directors Association. 2014 Dec;15(12):929-33.

36. Hung CY, Wu TJ, Wang KY, Huang JL, Loh EW, Chen YM, et al. Falls and atrial fibrillation in elderly patients. Acta Cardiologica Sinica. 2013 September;29(5):436-43.

37. Mlynarska A, Mlynarski R, Golba KS. Older age and a higher EHRA score allow higher levels of frailty syndrome to be predicted in patients with atrial fibrillation. Aging Male. 2017 Mar;20(1):23-7.

38. O'Caoimh R, Igras E, Ramesh A, Power B, O'Connor K, Liston R. Assessing the Appropriateness of Oral Anticoagulation for Atrial Fibrillation in Advanced Frailty: Use of Stroke and Bleeding Risk-Prediction Models. The Journal of frailty & aging. 2017 2017;6(1):46-52.

39. Polidoro A, Stefanelli F, Ciacciarelli M, Pacelli A, Di Sanzo D, Alessandri C. Frailty in patients affected by atrial fibrillation. Archives of Gerontology and Geriatrics. 2013 Nov-Dec;57(3):325-7.

40. Hess PL, Kim S, Piccini JP, Allen LA, Ansell JE, Chang P, et al. Use of Evidence-based Cardiac Prevention Therapy Among Outpatients with Atrial Fibrillation. American Journal of Medicine. 2013 Jul;126(7):625-+.

41. Bo M, Li Puma F, Martini MB, Falcone Y, Iacovino M, Grisoglio E, et al. Effects of oral anticoagulant therapy in older medical in-patients with atrial fibrillation: a prospective cohort observational study. Aging Clinical and Experimental Research. 2017 Jun;29(3):491-7.

42. Doucet J, Greboval-Furstenfeld E, Tavildari A, M'Bello L, Delaunay O, Pesque T, et al. Which parameters differ in very old patients with chronic atrial fibrillation treated by anticoagulant or aspirin? Antithrombotic treatment of atrial fibrillation in the elderly. Fundamental and Clinical Pharmacology. 2008 October;22(5):569-74.

43. Gullon A, Formiga F, Camafort M, Mostaza JM, Diez-Manglano J, Cepeda JM, et al. Baseline functional status as the strongest predictor of in-hospital mortality in elderly patients with non-valvular atrial fibrillation: Results of the NONAVASC registry. European journal of internal medicine. 2017 2017-Sep-24.

44. Magnani JW, Wang N, Benjamin EJ, Garcia ME, Bauer DC, Butler J, et al. Atrial Fibrillation and Declining Physical Performance in Older Adults The Health, Aging, and Body Composition Study. Circulation-Arrhythmia and Electrophysiology. 2016 May;9(5).

45. Nguyen TN, Cumming RG, Hilmer SN. The Impact of Frailty on Mortality, Length of Stay and Re-hospitalisation in Older Patients with Atrial Fibrillation. Heart Lung and Circulation. 2016 Jun;25(6):551-7.

46. Pilotto A, Gallina P, Copetti M, Pilotto A, Marcato F, Mello AM, et al. Warfarin Treatment and All-Cause Mortality in Community-Dwelling Older Adults with Atrial Fibrillation: A Retrospective Observational Study. Journal of the American Geriatrics Society. 2016 Jul;64(7):1416-24.

47. Frewen J, Finucane C, Rice C, Kearney P, Kenny RA, Harbison JA. The use of anticoagulation therapy in subjects with atrial fibrillation in the irish longitudinal study of ageing (TILDA). Cerebrovascular Diseases. 2012 May;33:822-3.

48. Podsiadlo D, Richardson S. The timed "Up & Go": a test of basic functional mobility for frail elderly persons. Journal of the American Geriatrics Society. 1991 Feb;39(2):142-8.

49. Rockwood K, Song X, MacKnight C, Bergman H, Hogan DB, McDowell I, et al. A global clinical measure of fitness and frailty in elderly people. CMAJ : Canadian Medical Association journal = journal de l'Association medicale canadienne. 2005 Aug 30;173(5):489-95.

50. Rolfson DB, Majumdar SR, Tsuyuki RT, Tahir A, Rockwood K. Validity and reliability of the Edmonton Frail Scale. Age and ageing. 2006 Sep;35(5):526-9.

51. Yao J-L, Fang J, Lou Q-Q, Anderson RM. A systematic review of the identification of seniors at risk (ISAR) tool for the prediction of adverse outcome in elderly patients seen in the emergency department. International Journal of Clinical and Experimental Medicine. 2015

52. Gill TM, Gahbauer EA, Han L, Allore HG. The role of intervening hospital admissions on trajectories of disability in the last year of life: prospective cohort study of older people. BMJ : British Medical Journal. 2015;350.

53. van den Ham HA, Klungel OH, Singer DE, Leufkens HGM, van Staa TP. Comparative Performance of ATRIA, CHADS2, and CHA2DS2-VASc Risk Scores Predicting Stroke in Patients With Atrial Fibrillation: Results From a National Primary Care Database. Journal of the American College of Cardiology. 2015 2015/10/27/;66(17):1851-9.

54. Clegg A, Bates C, Young J, Ryan R, Nichols L, Ann Teale E, et al. Development and validation of an electronic frailty index using routine primary care electronic health record data. Age Ageing. 2016 May;45(3):353-60.

55. Man-Son-Hing M, Nichol G, Lau A, Laupacis A. Choosing antithrombotic therapy for elderly patients with atrial fibrillation who are at risk for falls. Arch Intern Med. 1999 Apr 12;159(7):677-85.

56. Bibas L, Levi M, Touchette J, Mardigyan V, Bernier M, Essebag V, et al. Implications of Frailty in Elderly Patients With Electrophysiological Conditions. JACC: Clinical Electrophysiology. 2016 2016/06/01/;2(3):288-94.

57. Lacoin L, Lumley M, Ridha E, Pereira M, McDonald L, Ramagopalan S, et al. Evolving landscape of stroke prevention in atrial fibrillation within the UK between 2012 and 2016: a cross-sectional analysis study using CPRD. BMJ Open. 2017;7(9).

58. Turner G, Clegg A. Best practice guidelines for the management of frailty: a British Geriatrics Society, Age UK and Royal College of General Practitioners report. Age and Ageing. 2014;43(6):744-7.

59. Searle SD, Mitnitski A, Gahbauer EA, Gill TM, Rockwood K. A standard procedure for creating a frailty index. BMC geriatrics. 2008 Sep 30;8:24.

60. Robinson TN, Wu DS, Pointer L, Dunn CL, Cleveland JC, Jr., Moss M. Simple frailty score predicts postoperative complications across surgical specialties. American journal of surgery. 2013 Oct;206(4):544-50.
